# OCT4 induces EMT and promotes ovarian cancer progression by regulating the PI3K/AKT/mTOR pathway

**DOI:** 10.3389/fonc.2022.876257

**Published:** 2022-08-10

**Authors:** Weiwei Xie, Jun Yu, Yujia Yin, Xiaoqian Zhang, Xiaocui Zheng, Xipeng Wang

**Affiliations:** Department of Obstetrics and Gynecology, Xinhua Hospital, Shanghai Jiaotong University School of Medicine, Shanghai, China

**Keywords:** OCT4, ovarian cancer, proliferation, migration, invasion, AKT

## Abstract

**Background:**

Octamer-binding transcription factor 4 (OCT4) is a key stem cell transcription factor involved in the development of various cancers. The role of OCT4 in ovarian cancer (OC) progression and its molecular mechanism are not fully understood.

**Methods:**

First, immunohistochemistry (IHC) assays of ovarian benign cyst tissues, OC tissues, and omental metastatic tissues were performed to reveal OCT4 expression profiles. We knocked down OCT4 in two OC cell lines (SKOV3 and A2780) using a lentiviral vector and performed *in vitro* and *in vivo* experiments. OCT4 was knocked down to assess the proliferation, migration, and invasion of OC cells using CCK-8, colony formation, wound healing, and Transwell assays. In addition, the nude tumor mouse model was used for *in vivo* study. Mechanistically, we demonstrated that OCT4 influenced protein expression in the phosphoinositol 3-kinase (PI3K)/AKT/mTOR pathway and epithelial-mesenchymal transition (EMT)-related proteins by Western blotting and immunofluorescence (IF) assays. The interaction between OCT4 and p-AKT was further confirmed by coimmunoprecipitation (CoIP) assays. Importantly, AKT activation by its activator SC79 reversed the biological functions of OCT4 knockdown.

**Results:**

OCT4 expression was significantly upregulated in OC samples and metastatic tissues. OCT4 knockdown notably inhibited the proliferation, migration, and invasion of OC cells *in vitro* and *in vivo*. Moreover, the expression of p-PI3K, p-AKT, and p-mTOR was downregulated after OCT4 knockdown. An AKT agonist reversed the effect of OCT4 knockdown on OC cells. EMT in OC samples was enhanced by OCT4.

**Conclusions:**

Our study shows that OCT4 promotes the proliferation, migration, and invasion of OC cells by participating in the PI3K/AKT/mTOR signaling axis, suggesting that it could serve as a potential therapeutic target for OC patients.

## Background

Over the past four decades, cancer survival has improved for most cancers (1). Despite these advances, OC is the fifth most frequent cause of cancer-related death in women, with the highest mortality rate among gynecologic malignancies (2). The American Cancer Society estimates that 13,940 OC-related deaths (5% of all cancer cases) occurred in the United States in 2021 (3). Although this disease is highly curable in the early stage, there is a lack of effective screening options at the early stage, most patients present with stage III/IV disease, and more than 75% of women with advanced OC die from the disease (4). Cytoreductive surgery and combined platinum‐taxane chemotherapy have been considered the mainstay of therapy for decades (5). Nevertheless, emphasizing the importance of potential biomarkers for early diagnosis and timely specialist treatment is increasingly urgent.

OCT4, an octamer binding transcription factor 4, also known as the Pit1, Oct1/Oct2, and Unc86 (POU) domain, class 5 homeobox 1 (POU5F1) and OCT3, is a member of the POU family of transcription factors (TFs). POU TFs family proteins are involved in the regulation of pluripotency in mammals. The main function of OCT4 is to bind the octamer sequence motif (ATGCAAAT) to activate the expression of target genes (6). Gene expression, protein modification, stability, and activity of OCT4 are strictly regulated during embryonic development (7). OCT4 has been convincingly established as one of the most important transcription factors to maintain the self-renewal and pluripotency of embryonic stem cells (ESCs) (8), germline stem cells and embryonic cancer cells (ECCs) (9). OCT4 is undetectable in the cells of mature organisms, so its re-expression is closely related to tumor development and progression (10). Studies have shown that high OCT4 expression is significantly associated with decreased overall survival (OS) in patients with pancreatic cancer (11). In gastric cancer cells, OCT4 and SOX2 promote tumor proliferation, migration, invasion, and tumorigenicity, and the two genes may have synergistic effects to some extent (12). However, the role of OCT4 in tumorigenesis has not been confirmed.

The serine/threonine kinase AKT (also known as protein kinase B, PKB) is a central molecule in the PI3K/AKT/mTOR signaling pathway. It has gained attention because of its key regulatory functions in various cellular processes, especially in cell proliferation and tumor progression (13). Aberrantly activated PI3K/AKT signaling is mainly caused by its corresponding hosphatase and tensin homologue deleted on chromosome 10 (PTEN) deficiency, which is implicated in the pathogenesis of a variety of cancers (14). Various studies indicate that in ESCs and ECCs, OCT4 and AKT interact directly or indirectly at multiple levels to form an AKT–OCT4 regulatory circuit (15, 16).

In this study, IHC assays were performed to analyze the expression profile of OCT4 by using a tissue microarray (TMA) of our samples. Two cell lines (SKOV3 and A2780) were used to detect the function of OCT4 and its mechanism in OC progression. Our findings may provide new directions for the diagnosis and treatment of OC.

## Methods

### Patients and specimens

Ovarian TMAs were obtained from patients diagnosed between 2008 and 2018 at Xinhua Hospital, Shanghai Jiaotong University School of Medicine after obtaining the participants’ informed consent. OC samples verified by postoperative pathology were included in the study, while patients with other malignant tumors were excluded. The histopathological diagnosis, grade, and stage of OC were based on the International Federation of Gynecology and Obstetrics (FIGO) classification. This study was approved by the Ethics Committee of Xinhua Hospital.

### Cell lines and culture

All cell lines were purchased from the American-type culture collection (ATCC, USA) and cultured at 37°C in a 5% CO_2_ atmosphere. The OC cell lines SKOV3, A2780, OVCA433, SKOV3-IP1, HEY, HEY A8, ES-2, and OVCA429 were cultured in Dulbecco’s modified Eagle’s medium (DMEM, Gibco, USA) containing 10% fetal bovine serum (FBS, Gibco, USA), penicillin (100 U/ml) and streptomycin (100 ng/ml). Normal ovarian epithelial cell lines (IOSE80) culture method is the same as above. All cell lines were authenticated by STR profiling and tested regularly for mycoplasma contamination (last tested in November 2021).

### Viral transduction

For the generation of stable cell lines, lentiviruses containing control vectors (Control) and specific short hairpin RNAs (shRNAs) against OCT4 (OCT4-sh1/sh2) were obtained from Shanghai Genochemical Company (Shanghai, China). Lentiviruses were generated by co-transduction of HEK293T cells with a mixture of recombinant lentivirus vectors and pPACK Packaged Plasmid (System Biosciences) using Megatran reagent (Origene). Lentivirus shRNA vectors were constructed by cloning short hairpin RNA fragments into pSIH-H1-Puro (System Biosciences). Lentiviral vectors for gene knockdown were obtained by inserting amplified gene fragments into pCDH (System Biosciences). The sequences of the shRNAs for Control and OCT4 are listed in **Supplementary Table S1**.

### Western blotting assay

Cells were lysed in RIPA buffer, and lysates were separated by 6%-10% SDS–PAGE. Then, the proteins were transferred onto polyvinylidene fluoride membranes (Millipore, IPVH00010). After the membranes were blocked with 5% BSA for 2 h, they were incubated with primary antibodies overnight at 4°C and washed with 1×Tris-buffered saline with Tween 20 (TBST). Primary antibodies used in the study include anti-OCT4 (Abcam, ab181557), anti-PI3K (Cell Signaling Technology, 4249), anti-p-PI3K p85 (Tyr458)/p55 (Tyr199) (Cell Signaling Technology, 17366), anti-AKT (Cell Signaling Technology, 4691), anti-p-AKT (Ser473) (Cell Signaling Technology, 4060S), anti- mTOR (Cell Signaling Technology, 2983), anti-p-mTOR (Ser2448) (Cell Signaling Technology, 5536T), anti-E-Cadherin (Proteintech, 20874-1-AP), and anti-N-cadherin (Proteintech, 22018-1-AP). Anti-ACTIN (Proteintech, 20536-1-AP) and anti-GAPDH (Proteintech, 10494-1-AP) were used as internal standard. Subsequently, the membranes were incubated with a secondary HRP-conjugated antibody for 2 h at room temperature (RT) and washed with TBST. Finally, the signals were visualized by Immobilon Western Chemiluminescent HRP Substrate (Millipore, US). The antibodies used are listed in **Supplementary Table S2**.

### IF assay

Cells were fixed in 4% paraformaldehyde for 30 min, permeated with 2% Triton X-100 for 10 min, and sealed in 5% goat serum for 60 min at RT. Subsequently, the samples were incubated at RT with primary and secondary antibodies for 60 min. For IF, primary antibodies used in the study include anti-OCT4 (Proteintech, 11263-1-AP) and anti-p-AKT (Ser473) (Cell Signaling Technology, 4060S). The secondary antibodies conjugated with Alexa Fluor 594 (Molecular Probes, USA) were then restained at RT for 30 min. Nuclei were restained with 4′,6-diamidino-2-phenylindole dihydrochloride (DAPI, Life Technologies, USA) at RT for 5 min. IF signals were captured using a Leica SP5 confocal fluorescence microscope (Wetzlar, Frankfurt, Germany).

### IHC

TMAs were conducted using ovarian benign cyst tissues, OC tissues, matched omental metastatic tissues, and an OCT4 antibody (ab181557, Abcam). According to the dyeing depth, the intensity of staining was classified as weak, moderate, and strong. Protein expression was semiquantified using the histochemistry score (H-score), which was calculated as ∑ (PI × I) = (percentage of cells of weak intensity × 1) + (percentage of cells of moderate intensity × 2) + (percentage of cells of strong intensity × 3). PI represents the proportion of positive signal pixel area, I represents the coloring intensity. The final score was calculated as the average H-score of the duplicate TMA for each tissue type (17, 18).

### Cell Counting Kit (CCK)−8 proliferation assay

A CCK−8 (Beyotime, Shanghai, China, Cat# C0039) assay was used to determine OC cell viability. Transfected SKOV3 and A2780 cells (Control, OCT4-sh1 and OCT4-sh2) were digested with trypsin and re-suspended in serum-containing medium. Count the cells in the suspension using a cell counting board to calculate the number of cells per microliter of liquid. Divide the total number of cells by the number of cells per microliter, which is the volume of suspended cell solution that needs to be added. Cells were seeded in 96−well plates at a density of 2000 cells per well. After culture for 1, 2, 3, 4, and 5 days, 100 µl of medium containing 10 µl of CCK−8 reagent was added, and the cells were incubated at 5% CO_2_ and 37°C for 2 h. The absorbance was measured at a wavelength of 450 nm using a plate reader.

### Colony formation assay

Transfected SKOV3 and A2780 cells (Control, OCT4-sh1 and OCT4-sh2) were digested with trypsin and re-suspended in serum-containing medium. Count the cells in the suspension using a cell counting board to calculate the number of cells per microliter of liquid. Divide the total number of cells by the number of cells per microliter, which is the volume of suspended cell solution that needs to be added. SKOV3 and A2780 cells were cultured in 6-well plates at 1000 cells per well. They were cultured at 5% CO_2_ and 37°C for at least 7 days. The colonies were stained with 4% paraformaldehyde for 20 min and 0.2% crystal violet for 15 min and counted.

### Wound healing assay

Transfected SKOV3 and A2780 cells (Control, OCT4-sh1 and OCT4-sh2) were digested with trypsin and re-suspended in serum-containing medium. Count the cells in the suspension using a cell counting board to calculate the number of cells per microliter of liquid. Divide the total number of cells by the number of cells per microliter, which is the volume of suspended cell solution that needs to be added. Cells were seeded in 6-well plates at 9×10^5^ cells per well as confluent monolayers. A single layer was wounded in the middle of the well with a standard pipette tip. Subsequently, the scratches were washed with phosphate-buffered saline (PBS) to remove cellular debris. After incubation for 24 h, the area of the cell-free wound was examined with microscopy.

### Migration and invasion assays

Transfected SKOV3 and A2780 cells (Control, OCT4-sh1 and OCT4-sh2) were digested with trypsin and re-suspended in serum-free medium. Count the cells in the suspension using a cell counting board to calculate the number of cells per microliter of liquid. Divide the total number of cells by the number of cells per microliter, which is the volume of suspended cell solution that needs to be added. Cell migration and invasion were evaluated using a Transwell chamber (8 μm pore; Corning, 3422). Cell suspensions (6 × 10^4^ cells) in serum-free DMEM were added to the upper chamber (for migration assays) or the chamber precoated with Matrigel (for invasion assays). DMEM containing 10% FBS was added to the lower chamber. After incubation for 16 h, the cells that invaded or migrated to the lower chamber were stained with 0.2% crystal violet for 15 min. Five random images of different fields of vision under a microscope (Olympus) were captured, and the number of migrated or invaded cells was counted.

### Immunoprecipitation (IP)

IP was performed using the Pierce Crosslink Immunoprecipitation kit according to the manufacturer’s protocol. Briefly, 10 μg antibody was covalently crosslinked with agarose A/G beads using dextran sodium sulfate (DSS) and incubated overnight with 1 mg total cell lysates at 4°C. Primary antibodies used in the study include anti-OCT4 (Abcam, ab181557) and anti-p-AKT (Ser473) (Cell Signaling Technology, 4060S). The antigen was eluted and subjected to SDS-PAGE.

### Immunoblotting (IB)

The cell lines were separated by 8-12% SDS-PAGE and electrotransferred to 0.2 µm nitrocellulose membranes. After blocking in bovine serum albumin (BSA) for 1 h, the membranes were incubated with the appropriate primary antibodies at 4°C overnight, followed by secondary antibody.

### Quantitative real-time PCR (qRT–PCR)

Total RNA from cells was extracted using TRIzol reagent (Invitrogen, Carlsbad, CA, USA), reverse-transcribed into cDNA, and subjected to PCR using the PrimeScript RT–PCR kit (Takara, Japan). Glyceraldehyde-3-phosphate dehydrogenase (GAPDH) was used as an internal control for CD49f mRNA. Three independent experiments were performed. The final analysis was calculated by 2^-ΔΔCT^ relative quantification, where ΔΔCT=ΔCT treatment-ΔCT control and ΔCT=CT target gene-CT GAPDH. The primer sequences are listed in **Supplementary Table S3**.

### Animal tumor model

Animal care and experimental procedures were performed following Guidelines for Animal Experiments and were approved by the Animal Care and Use Committee of Xinhua Hospital in our study. Five-week-old female BALB/c nude mice (Department of Laboratory Animals, Xinhua Hospital) were randomly divided into different groups (n=5) for subcutaneous injection with A2780-control, A2780-OCT4-sh1, and A2780-OCT4-sh2 cells (2×10^6^ cells for each mouse). The tumor volume was calculated using the following formula: volume =width^2^×length×0.52. After subcutaneous injection, tumor development was assessed weekly. Five weeks later, the tumors in each group were harvested after euthanasia, and the weights of the tumors and the number of metastatic organs were recorded. Tumor tissues and metastatic organs were fixed with 4% neutral buffered formalin for frozen section preparation.

### Statistical analysis

The data in this study were calculated using SPSS 24.0 software (SPSS Inc., Chicago, IL, USA) and GraphPad Prism (Version 8.0, San Diego, CA, USA) and are presented as the mean ± standard deviation (SD). Student’s t test, two-way ANOVA, and chi-square tests were used for comparisons between groups. Each experiment was repeated at least trice. A probability less than 0.05 was considered significant. Kaplan–Meier survival analysis was used to evaluate the correlation between clinical outcome and gene expression, and the P value was calculated by the log-rank test.

## Results

### OCT4 expression was significantly upregulated and was closely related to clinicopathological features in OC

**Figure 1:**
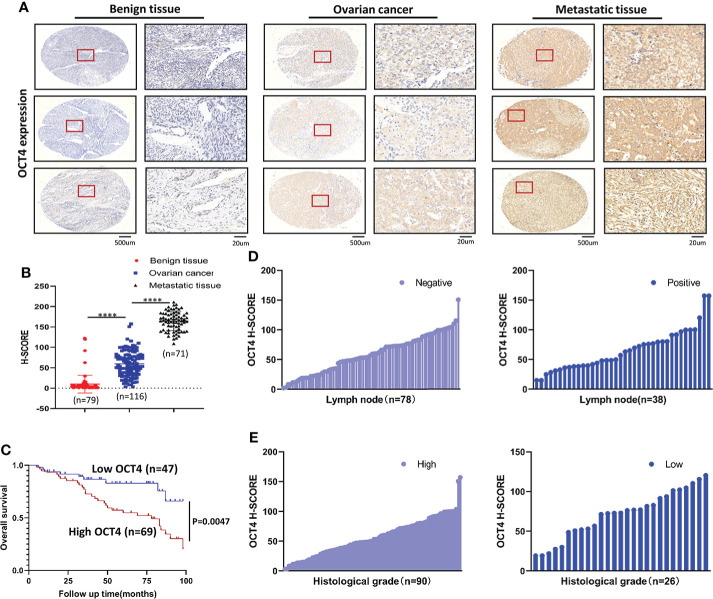
OCT4 IHC staining and survival analysis **(A)** Representative IHC staining of OCT4 in benign ovarian cyst tissues, OC tissues, and matched omental metastatic tissues. **(B)** Quantification of OCT4 H-score in different tissues. **(C)** OS of OC patients with low/high OCT4 H-score. **(D)** Expression rate of OCT4 in OC with or without lymph node metastasis. **(E)** Expression rate of OCT4 in high/low histological grade OC. (Data were expressed as the mean ± SD. Significance was calculated using Student’s t-test. ****, P<0.0001.)

To determine whether OCT4 expression is correlated with OC progression, we collected ovarian benign cyst tissues (n=79), OC primary tumor tissues (n=116), and OC metastatic tissues in the greater omentum (n=71) and examined OCT4 expression by using IHC. The OCT4 H-score of the primary and metastatic tissues was significantly higher than that of the ovarian benign tissues (**Figures 1A, B**). In addition, OCT4 expression was further increased in metastatic tissues compared with primary OC tissues (**Figures 1A, B**). To investigate the clinical significance of OCT4 in OC, we collected the medical history of 116 OC patients and analyzed the association between OCT4 expression and the clinicopathologic characteristics of OC patients (**Table 1**). The boundary between high-level and low-level expression of OCT4 was the median H-score. As shown in **Table 1**, OCT4 expression was significantly correlated with histological grade and lymph node metastasis (**Figures 1D, E**), while there was no significant association between OCT4 expression and age, FIGO stage, or histological subtype. Following information about patient outcomes, survival analysis showed that high expression of OCT4 was significantly associated with poorer OS (**Figure 1C** **)**. Taken together, these data suggested that upregulated expression of OCT4, as an independent prognostic factor, had a significant correlation with poor prognosis of OC and may contribute to OC progression.

**Table 1:** Correlations between OCT4 expression and clinicopathologic parameters in 116 OC patients.

Clinicopathologicalparameter	No. of patients	OCT4 expression		P value
	(n=116)	Low (n=47, %)	High (n=69, %)	
Age (years)				0.562
<50	43	16 (37.21%)	27 (62.79%)	
≥50	73	31 (42.47%)	42 (57.53%)	
FIGO stage				0.296
I + II	66	30 (45.45%)	36 (54.55%)	
III + IV	50	17 (34.00%)	33 (66.00%)	
Histological subtype				0.074
EOC	103	34 (33.01%)	69 (66.99%)	
NEOC	13	13 (100%)	0 (0%)	
Histological grade				**0.004**
Low	26	12 (46.15%)	14 (53.85%)	
High	90	35 (38.89%)	55 (61.11%)	
Lymph node				**0.019**
Positive	38	17 (44.74%)	21 (55.26%)	
Negative	78	30 (38.46%)	48 (61.54%)	

P values in bold indicate that they are statistically significant. We have removed the remaining bold and re-uploaded the table.

### OCT4 protein expression was upregulated in the OC cell lines SKOV3 and A2780

**Figure 2:**
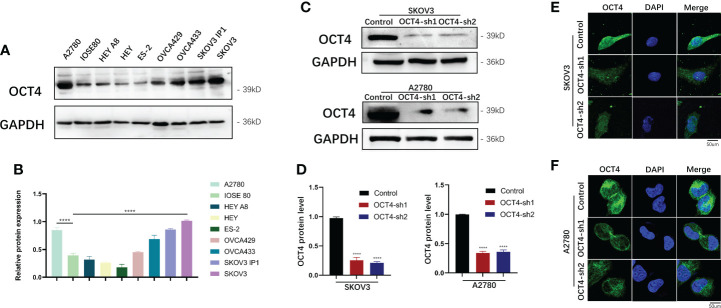
Expression of OCT4 in OC cell lines **(A, B)** OCT4 protein expression in OC cell lines compared with normal ovarian epithelial cell lines (IOSE80). **(C, D)** OCT4 protein expression in SKOV3 and A2780 cells transfected with shRNAs targeting OCT4 by Western blotting assay. **(E, F)** The above expression was verified by IF analysis. (Data were expressed as the mean ± SD. Significance was calculated using Student's t-test. ****, P<0.0001.)

Compared with that in normal ovarian epithelial cell lines (IOSE80), OCT4 was highly expressed in OC cell lines (SKOV3, A2780, OVCA433, and SKOV3-ip1), whereas its expression was relatively low in HEY, HEY A8, ES-2, and OVCA429 cells (**Figures 2A, B**). OCT4 expression was particularly high in two invasive cell lines, SKOV3 and A2780, compared with other cell lines. To further explore the regulatory role of OCT4 in OC, we constructed stable OCT4 knockdown cell lines (OCT4-sh1 and OCT4-sh2) and control vector cell lines (Control) by lentiviral transduction in SKOV3 and A2780 cells, which was verified by Western blotting assays (**Figures 2C, D**) and IF analysis (**Figures 2E, F**).

### Knockdown of OCT4 inhibited the proliferation of OC cells

**Figure 3:**
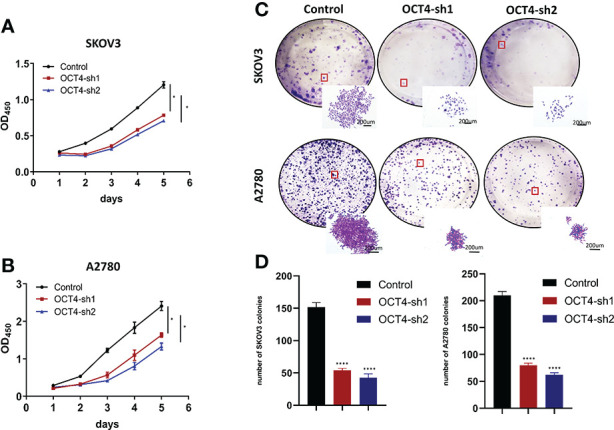
Knockdown of OCT4 inhibited the proliferation of OC cells **(A, B)** In SKOV3 and A2780 cell lines, cell viability was measured by CCK-8 assay. **(C, D)** Colony formation assays and statistical analysis in SKOV3 and A2780 cell lines. (Data were expressed as the mean ± SD. Significance was calculated using Student's t-test. *P<0.05, ****P<0.0001).

Next, we tested the effect of OCT4 knockdown on proliferation by CCK-8 assays and found that OCT4 knockdown significantly reduced cell growth (**Figures 3A, B**). Colony formation assays indicated that OCT4 knockdown decreased the size and number of colonies of SKOV3 and A2780 cells (**Figures 3C, D**).

### Knockdown of OCT4 inhibited the migration and invasion of OC cells

**Figure 4:**
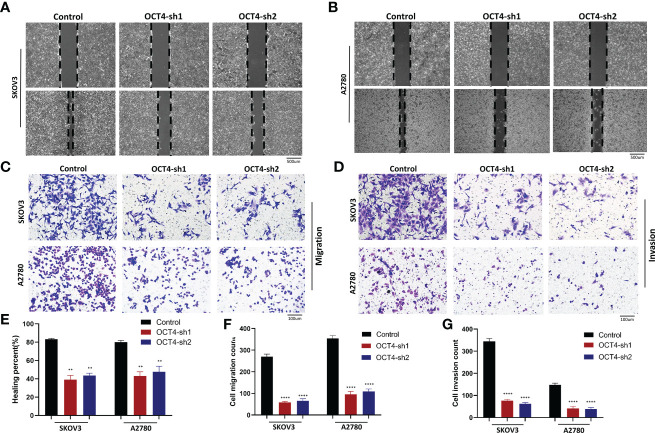
Knockdown of OCT4 inhibited the migration and invasion of OC cells **(A, B)** In two cell lines, wound healing assays indicated the percent of wounds closed. **(C, D)** Transwell assays were used to measure the migration and invasion of two cell lines. **(E–G)** Quantification of cell migration and invasion was measured by wound healing or Transwell assays. (Data were expressed as the mean ± SD. Significance was calculated using Student’s t-test. **P<0.01, ****P<0.0001).

By performing wound healing assays, we found that the flattening and spreading range was decreased in the SKOV3-OCT4-sh1/2 and A2780-OCT4-sh1/2 cells, respectively, compared with the controls (**Figures 4A, B, E**). Transwell assays revealed that cell migration and invasion were significantly inhibited in the OCT4 knockdown cells compared with the control cells (**Figures 4C, D, F, G**).

### Knockdown of OCT4 inhibited the PI3K/AKT/mTOR pathways and EMT in OC cells

**Figure 5:**
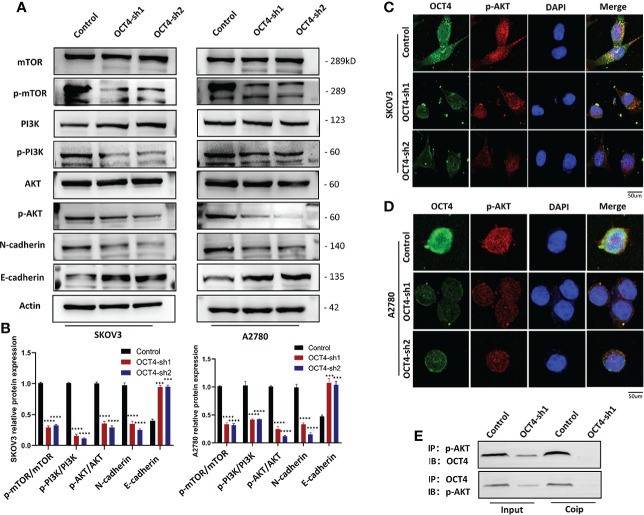
OCT4 influenced the PI3K/AKT/mTOR signaling pathway in OC cells. **(A, B)** Western blotting was used to measure representative proteins and their relative expression levels in the PI3K/AKT/mTOR pathway. In addition, OCT4 knockdown inhibited the expression of the EMT pathway. **(C, D)** IF assays detected the relationship between OCT4 and p-AKT in OC cell lines. **(E)** CoIP of OCT4 with p-AKT in A2780 cell lines. The input represents the total protein extract used in IP. IB, immunoblotting; IP, immunoprecipitation. (Data were expressed as the mean ± SD. Significance was calculated using Student's t-test. ***P<0.001, ****P<0.0001).

The PI3K/AKT/mTOR signaling pathway is one of the most frequently identified pathways in human cancer and plays a key role in driving tumor initiation and progression (19). To explore the downstream signaling pathway regulated by OCT4, we used Western blotting to determine the association between OCT4 and the PI3K/AKT/mTOR signaling pathway. OCT4 knockdown inhibited the accumulation of p-PI3K, p-AKT, and p-mTOR proteins, suggesting that OCT4 may regulate the progression of OC through the PI3K/AKT/mTOR signaling pathway (**Figures 5A, B**). To look for evidence of phosphorylation of AKT by OCT4, we examined SKOV3 and A2780 cell lines and found that endogenous OCT4 colocalized with phosphorylated AKT (AKT-pS473) in the nucleus (**Figures 5C, D**). The interaction between OCT4 and p-AKT was further confirmed by CoIP assays (**Figure 5E**). Remarkably, their interaction was substantially decreased after OCT4 knockdown (**Figure 5E**). Loss of E-cadherin gene expression promotes dysfunction of the cell connection system, leading to invasion and metastasis of cancer cells, which has been considered the most important feature of EMT. Western blotting of the EMT markers E-cadherin and N-cadherin in the SKOV3-OCT4-sh1/sh2 and A2780-OCT4-sh1/sh2 cell lines showed that OCT4 knockdown inhibited EMT (**Figures 5A, B**).

A novel connection between CD49f (integrin α6) and the PI3K/AKT/mTOR signaling pathway in tumor cells has been identified (20). CD49f is required for the migration and adhesion of endothelial progenitor cells, and OCT4 and SOX2 were found to be recruited to its promoter region (21). Overexpression of CD49f resulted in increased phosphorylation levels of the PI3K/AKT pathway and GSK3β (20). Taken together, these results showed that OCT4 and the PI3K/AKT pathways can be connected through CD49f. Through qRT–PCR, we found that CD49f expression was significantly lower in SKOV3-OCT4-sh1/sh2 and A2780-OCT4-sh1/sh2 cell lines (**Figure S1A–B**).

### AKT agonist reversed the effect of OCT4 knockdown on OC cells

**Figure 6:**
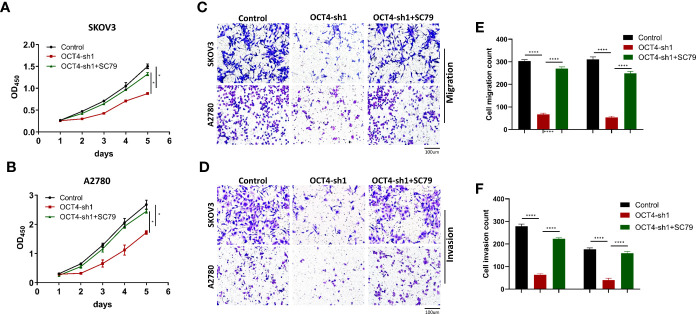
OCT4 modulated proliferation, migration and invasion by activating the AKT signaling pathway in OC cells. **(A, B)** In SKOV3 and A2780 cell lines, CCK-8 assay data showed that SC79 treatment rescued the inhibition of proliferation by OCT4 knockdown. **(C–F)** Transwell assays indicated that the suppression of migration and invasion ability by OCT4 knockdown was rescued after SC79 treatment. (Data were expressed as the mean ± SD. Significance was calculated using Student’s t-test. *P<0.05, ****P<0.0001.)

According to previous studies, the phosphorylation levels of PI3K, AKT and mTOR decreased significantly after OCT4 knockdown. To further investigate the mechanism of OCT4 activation of the PI3K/AKT/mTOR pathway in OC cells, the AKT phosphorylation agonist SC79 (5 μg/mL) (22) was used to treat SKOV3-OCT4-sh1 and A2780-OCT4-sh1 cells. CCK-8 assays showed that 10 µM SC79 could rescue the viability of SKOV3 and A2780 cells after OCT4 knockdown (**Figures 6A, B**). Transwell assays demonstrated that activation of the AKT pathway by SC79 exposure abolished OCT4 knockdown induced suppression of cell migration and invasion (**Figures 6C–F**).

### Knockdown of OCT4 inhibited the proliferation and metastasis of OC *in vivo*

**Figure 7:**
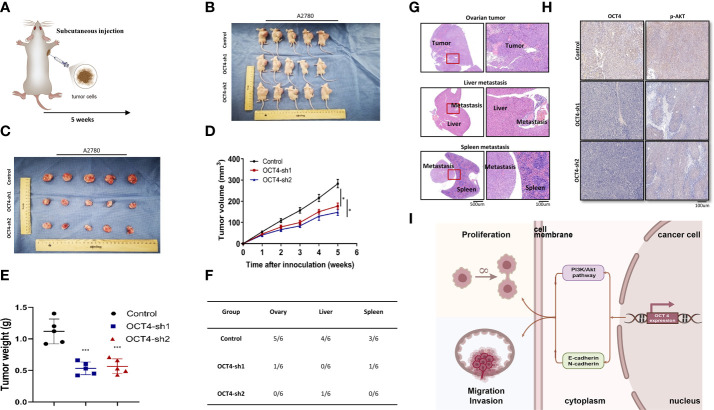
The effect of OCT4 knockdown on the proliferation and metastasis of OC in vivo. **(A)** The subcutaneous injection model of nude mice. **(B, C)** Size of tumor-bearing nude mice in different groups. **(D)** Tumor volume was measured weekly after injecting tumor cells. **(E)** Quantification of tumor weight. **(F)** Analysis of the location and number of metastases in tumor-bearing mice of different groups. **(G)** HE staining of ovarian neoplasms and metastatic tissues. **(H)** IHC assays of OCT4 and p-AKT in mouse tumors. **(I)** Model diagram showing the role of OCT4 and the PI3K/AKT/mTOR pathways as well as the EMT pathways regulating the proliferation and metastasis of OC cells. (Data were expressed as the mean ± SD. Significance was calculated using Student’s t-test. *P<0.05, ***P<0.001.).

To further confirm the role of OCT4 *in vivo*, we established a xenograft model in nude mice using the A2780 cell line (**Figure 7A**). The model results showed that tumor growth was significantly inhibited by OCT knockdown (**Figures 7B, C**). OCT4 knockdown reduced the volume and weight of A2780 cell-derived tumors (**Figures 7D, E**). The mice inoculated with A2780-OCT4-Control developed more metastases, while fewer metastases were observed in the A2780-OCT4-sh1/sh2 mouse tumor models (**Figure 7F**). Hematoxylin and eosin (HE) staining was performed on the right ovarian, liver, and spleen metastases of the tumor-bearing mice (**Figure 7G**). We conducted an IHC assay on mouse tumors and found that the OCT4-sh1/sh2 group had reduced expression levels of p-AKT (**Figure 7H**). Finally, we identified the roles of OCT4 and the PI3K/AKT/mTOR pathways as well as the EMT pathways in the regulation of proliferation and metastasis of OC (**Figure 7I**).

## Discussion

OCT4 promoted the formation and progression of cancer and was associated with poor clinical outcomes (23–26). OCT4 expression has been detected in various types of malignant neoplasms, and increased expression levels are associated with advanced tumor grade, metastatic formation, and survival rate, underscoring the clinical relevance of OCT4 (25). Several studies have shown that OCT4 expression is upregulated in head and neck squamous carcinoma (23), bladder cancer (24, 27), lung cancer (25), breast cancer (28), oral squamous cell carcinoma (29), and cervical cancer (30). These findings suggest the role of OCT4 in tumorigenesis. However, it is not clear which specific signaling pathways mediate the regulation of OCT4 expression.

The core focus of tumor research has been to detect tumor markers and identify directions for targeted therapies, especially after the tremendous efforts of The Cancer Genome Atlas (TCGA) project sequencing. Abnormal expression of genes is believed to be involved in cancer progression and metastasis. Our current understanding of OCT4 function supports its importance in tumorigenesis, where its presence is associated with poorer prognosis in most cancer types. Increased expression of OCT4 has been reported to be associated with the differentiation of pancreatic cancer, while OCT4 knockdown inhibits the invasion and growth of pancreatic cancer cell by inhibiting AKT pathway mediated proliferating cell nuclear antigen (PCNA) and matrix metalloproteinase-2 (MMP-2) expression (31). However, the mechanism and pathway of OCT4 in OC remain unknown.

In this study, we performed IHC staining and survival analysis on primary and metastatic OC tissues to prove that OCT4 can be regarded as a prognostic risk factor to induce OC progression and metastasis. OCT4 was significantly elevated in OC tissues and cell lines. We constructed two OC cell lines with OCT4 knockdown using stable lentivirus strains. Through CCK-8, colony formation, wound healing, and Transwell assays, we found that OCT4 significantly promoted cell proliferation and invasion *in vitro*. Next, we established a xenograft model in nude mice using A2780 cells to demonstrate that OCT4 promotes tumor progression and metastasis *in vivo*.

PI3K is one of the most frequently altered pathways in human malignancies, controlling most characteristics of cancer, including cell proliferation, migration, metastasis, and survival (32). AKT is a serine-threonine kinase, a known PI3K target that regulates numerous downstream target genes (33). To further investigate whether the PI3K/AKT/mTOR signaling pathway was involved in the oncogenic mechanism of OCT4 in OC, we evaluated the relationship between OCT4 and p-PI3K, p-AKT, or p-mTOR. Compared with the control group, the phosphorylation levels of PI3K, AKT and mTOR were significantly reduced after OCT4 knockdown, suggesting that OCT4 promoted the activation of the PI3K/AKT/mTOR signaling pathway in OC. CoIP experiments showed the interaction between OCT4 and p-AKT. OCT4 can also activate the AKT pathway through the controlled factor CD49f. To prove that OCT4 knockdown inhibits OC cell proliferation, migration, and invasion and is related to decreased AKT phosphorylation, we used the AKT phosphorylation activator SC79 *in vitro*. The results showed that activation of AKT could rescue the effects of OCT4 knockdown on OC cells, indicating that OCT4 was activated through the AKT pathway. However, the exact mechanism by which OCT4 interacts with the well-known signaling pathway remains to be defined. Based on current research, speculations can be made. PI3K recruits AKT to the plasma membrane to cause a conformational change, which leads to phosphorylation at the Thr308 site of AKT by phosphoinositide kinase 1 (PDK1) and the Ser473 site by mTORC2 phosphorylation (34, 35). AKT is dephosphorylated by the tumor suppressor PTEN and PH-domain leucine-rich protein phosphatase (PHLPP) (36). Interestingly, concurrent elevated AKT activation and OCT4 expression have been reported in some tumor cells and are related to chemotherapeutic drug resistance (37). This will have vast research prospects.

EMT is considered a pathological process that leads to tumor progression and is related to invasion and metastasis (38, 39). Heterogeneous cells may undergo oncogenic EMT, resulting in the loss of cell–cell polarity and adhesion, decreased epithelial protein expression, increased migration and invasion, and enhanced diffusion of primary tumors (40). In nasopharyngeal carcinoma, OCT4 is located in the anterior area of tumor invasion and is significantly associated with various invasive behaviors and EMT (41). In our research, we detected increased E-cadherin and reduced N-cadherin expression in OCT4 knockdown cell lines. OCT4 induces EMT events in OC, indicating the developing process of malignant cellular behavior. EMT is a complex biological process that involves multiple regulatory signaling pathway. In addition to the classical Wnt/β-catenin pathway, the PI3K/AKT signaling pathway has been reported that it can directly or through cooperation with other signaling pathways affect the EMT to induce tumor aggressiveness (42). The PI3K/AKT signaling pathway mediates the process of EMT and may serve as a potential target for the prevention and treatment of metastatic tumors (43, 44). However, in our study, there is a lack of direct evidence that PI3K is an important way to regulate the main marker of EMT. The limitation about the mechanism of EMT process by OCT4 should be noted. Its detailed mechanism needs to be clarified by using PI3K/AKT inhibitors in our further study. In addition, whether OCT4 induces OC cells migration and invasion by promoting EMT process needs to elucidate the underlying mechanisms.

## Conclusion

OCT4 expression is upregulated in OC, but this is the first study to show that OCT4 promotes proliferation and metastasis by affecting the PI3K/AKT/mTOR pathway in OC cells. OCT4 promotes the EMT process. Our research shows that OCT4, as an independent predictor, is significantly associated with poor prognosis and contributes to the progression of OC.

## Data availability statement

The original contributions presented in the study are included in the article/**Supplementary Material**. Further inquiries can be directed to the corresponding author.

## Ethics statement

The studies involving human participants were reviewed and approved by The Institutional Research Ethics Committee of Xinhua Hospital. The patients/participants provided their written informed consent to participate in this study. The animal study was reviewed and approved by The Ethics Committee of Xinhua Hospital, Shanghai Jiaotong University School of Medicine.

## Author contributions

WX performed the experiments, analyzed the data, and wrote the manuscript. JY developed the structure of the article and made significant revisions to the manuscript. YY designed the figures. XQZ and XCZ researched appropriate references. XW conceived and coordinated the project. All authors contributed to the article and approved the submitted version.

## Funding

This work was supported by the National Natural Science Foundation of China (81930064, 81874103 to XW).

## Acknowledgments

We thank all the authors and our colleagues for their product suggestions and discussions.

## Conflict of interest

The authors declare that the research was conducted in the absence of any commercial or financial relationships that could be construed as a potential conflict of interest.

## Publisher’s note

All claims expressed in this article are solely those of the authors and do not necessarily represent those of their affiliated organizations, or those of the publisher, the editors and the reviewers. Any product that may be evaluated in this article, or claim that may be made by its manufacturer, is not guaranteed or endorsed by the publisher.

## Glossary

**Table:**

OCT4	octamer-binding transcription factor 4
OC	ovarian cancer
IHC	immunohistochemistry
PI3K	phosphoinositol 3-kinase
EMT	epithelial-mesenchymal transition
IF	immunofluorescence
CoIP	Coimmunoprecipitation
POU	Pit1, Oct1/Oct2, and Unc86
POU5F1	POU class 5 homeobox 1
TFs	transcription factors
ESCs	embryonic stem cells
ECCs	embryonic cancer cells
OS	overall survival
PKB	protein kinase B
PTEN	hosphatase and tensin homologue deleted on chromosome 10
TMA	tissue microarray
FIGO	International Federation of Gynecology and Obstetrics
ATCC	American-type culture collection
DMEM	Dulbecco’s modified Eagle’s medium
FBS	fetal bovine serum
shRNAs	short hairpin RNAs
TBST	Tris-buffered saline with Tween20
RT	room temperature
DAPI	4′,6-diamidino-2-phenylindole dihydrochloride
H-score	histochemistry score
CCK8	Cell Counting Kit 8
PBS	phosphate-buffered saline
GAPDH	glyceraldehyde-3-phosphate dehydrogenase
qRT–PCR	Quantitative real-time PCR
IP	immunoprecipitation
DSS	dextran sodium sulfate
IB	immunoblotting
BSA	bovine serum albumin
SD	standard deviation
HE	hematoxylin and eosin
TCGA	The Cancer Genome Atlas
PCNA	pathway mediated proliferating cell nuclear antigen
MMP-2	matrix metalloproteinase–2
PDK1	phosphoinositide kinase 1
PHLPP	PH-domain leucine-rich protein phosphatase
