# Supplementary figures and images for: OCT4 induces EMT and promotes ovarian cancer progression by regulating the PI3K/AKT/mTOR pathway
Source: Front Oncol. 2022 Aug 10;12:876257. doi: 10.3389/fonc.2022.876257 (PMC9399417; doi:10.3389/fonc.2022.876257)

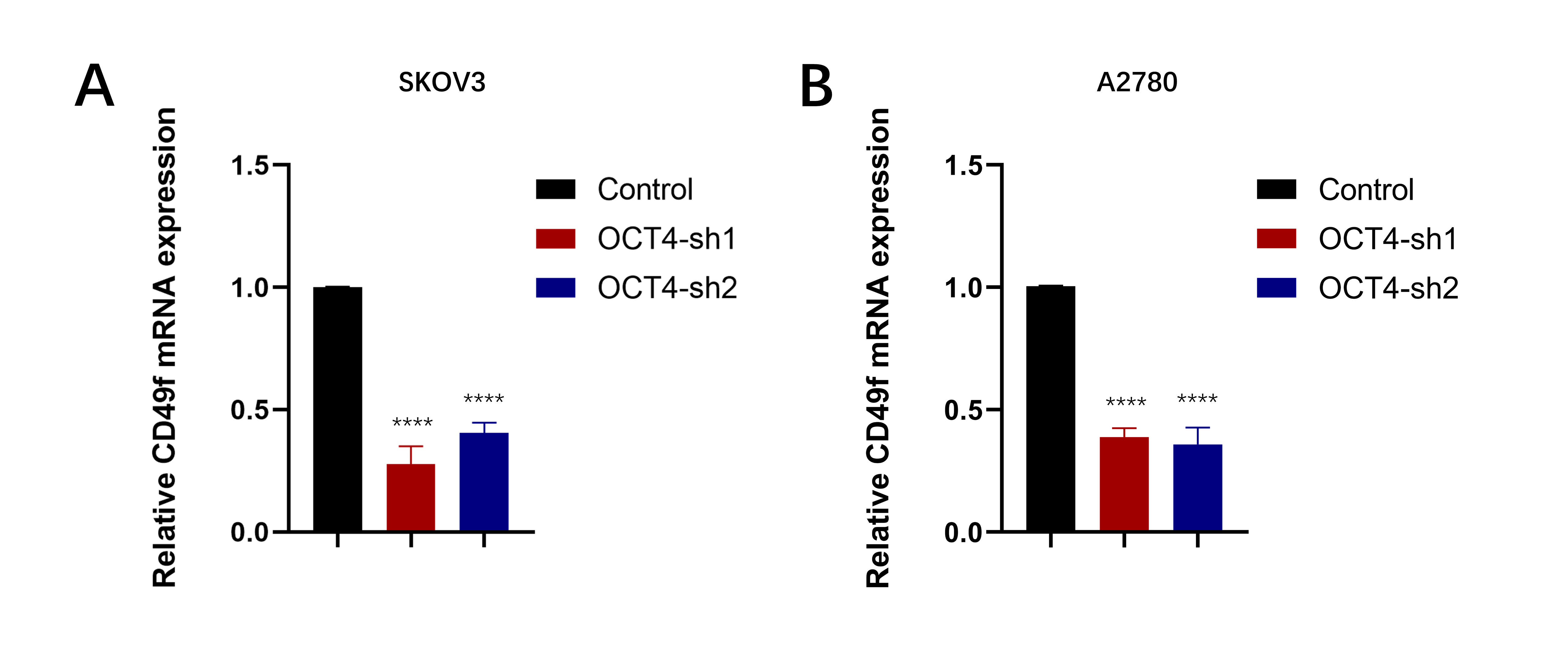

Supplement: Supplementary file 1 [file Image_1.tif]
