# Supplementary material for: OCT4 induces EMT and promotes ovarian cancer progression by regulating the PI3K/AKT/mTOR pathway
Source: Front Oncol. 2022 Aug 10;12:876257. doi: 10.3389/fonc.2022.876257 (PMC9399417; doi:10.3389/fonc.2022.876257)
Supplement: Supplementary file 2 [file Table_1.docx]

Supplementary Table 1. OCT4 shRNA sequence

| shRNA | sequence |
| --- | --- |
| Control sense | TTCTCCGAACGTGTCACGT |
| Control antisense | ACGTGACACGTTCGGAGAA |
| shRNA1 sense | GGCCACACGTAGGTTCTTGAA |
| shRNA1 antisense | TTCAAGAACCTACGTGTGGCC |
| shRNA2 sense | AGGTTCTTGAATCCCGAATGG |
| shRNA2 antisense | CCATTCGGGATTCAAGAACCT |
