# Supplementary material for: OCT4 induces EMT and promotes ovarian cancer progression by regulating the PI3K/AKT/mTOR pathway
Source: Front Oncol. 2022 Aug 10;12:876257. doi: 10.3389/fonc.2022.876257 (PMC9399417; doi:10.3389/fonc.2022.876257)
Supplement: Supplementary file 3 [file Table_2.docx]

Supplementary Table 2. Primary antibodies

| Primary antibodies | SOURCE | IDENTIFIER | Application |  |
| --- | --- | --- | --- | --- |
| OCT4 | Abcam | Cat# ab181557 | WB (1:1000) & IHC (1:1000) & IP (1:50) |  |
| OCT4 | Proteintech | Cat# 11263-1-AP | IF (1:200) |  |
| ACTIN | Proteintech | Cat# 20536-1-AP | WB (1:2000) |  |
| GAPDH | Proteintech | Cat# 10494-1-AP | WB (1:10000) |  |
| PI3K | Cell Signaling Technology | Cat# 4249 | WB (1:1000) |  |
| p-PI3K p85 (Tyr458)/p55 (Tyr199) | Cell Signaling Technology | Cat# 17366 | WB (1:1000) |  |
| E-Cadherin | Proteintech | Cat# 20874-1-AP | WB (1:5000) |  |
| N-cadherin | Proteintech | Cat# 22018-1-AP | WB (1:4000) |  |
| AKT | Cell Signaling Technology | Cat# 4691 | WB (1:1000) |  |
| p-AKT (Ser473) | Cell Signaling Technology | Cat# 4060S | WB (1:2000) & IF (1:500) & IP (1:50) |  |
| mTOR | Cell Signaling Technology | Cat# 2983 | WB (1:1000) |  |
| p-mTOR (Ser2448) | Cell Signaling Technology | Cat# 5536T | WB (1:1000) |  |
