# Supplementary material for: OCT4 induces EMT and promotes ovarian cancer progression by regulating the PI3K/AKT/mTOR pathway
Source: Front Oncol. 2022 Aug 10;12:876257. doi: 10.3389/fonc.2022.876257 (PMC9399417; doi:10.3389/fonc.2022.876257)
Supplement: Supplementary file 4 [file Table_3.docx]

Supplementary Table 3. Primer sequence

| Gene | Primer sequence | Application |
| --- | --- | --- |
| CD49f | Forward Primer: GGCGGTGTTATGTCCTGAGTC | qRT-PCR |
|  | Reverse Primer: AATCGCCCATCACAAAAGCTC |  |
| GAPDH | Forward Primer: GGAGCGAGATCCCTCCAAAAT | qRT-PCR |
|  | Reverse Primer: GGCTGTTGTCATACTTCTCATGG |  |
